# Reporting of Statistical Inference in Abstracts of Major Cancer Journals, 1990 to 2020

**DOI:** 10.1001/jamanetworkopen.2022.18337

**Published:** 2022-06-23

**Authors:** Andreas Stang, Börge Schmidt

**Affiliations:** 1 Institute of Medical Informatics, Biometry, and Epidemiology, University Hospital of Essen, Essen, Germany; 2 Department of Epidemiology, Boston University School of Public Health, Boston, Massachusetts

## Abstract

**Importance:**

Since the 1990s, reporting guidelines have developed that uniformly require authors to report a measure of precision (confidence intervals [CIs]) in addition to effect size.

**Objective:**

To investigate the time trend of statistical inference and statistical reporting style in abstracts of major cancer journals.

**Design, Setting, and Participants:**

This cross-sectional study reviewed all abstracts published between January 1, 1990, and December 31, 2020, in 10 high-ranking cancer journals (*Lancet Oncology*, *Journal of Clinical Oncology*, *Cancer Discovery*, *Cancer Cell*, *JAMA Oncology*, *Annals of Oncology*, *Molecular Cancer*, *Journal of Thoracic Oncology*, *Journal of the National Cancer Institute*, and *Trends in Cancer*) using a previously validated computerized algorithm to search the PubMed database. For the time trend analyses, 2 journals with only a few years of existence (*JAMA Oncology* and *Trends in Cancer*) were excluded.

**Exposures:**

Calendar year, journal, and type of abstract (randomized clinical trial or other).

**Main Outcomes and Measures:**

Proportions of abstracts containing CIs, *P* values without CIs, and qualitative expressions of statistical significance only were compared over time among journals.

**Results:**

Overall, 24 034 of 42 509 abstracts (56.5%) contained statistical inference. Reporting of CIs increased over time in 5 of 8 journals. From 2016 to 2020, the most prevailing statistical reporting style was the presentation of CIs (3070 of 4895 [62.7%]). However, the proportion of abstracts reporting statistical inference based solely on the terms *significant* or *nonsignificant* was still 1195 of 4895 (24.4%) during this period and was most prevalent among basic science–oriented cancer journals (eg, 63 of 66 [95.5%] in *Cancer Cell*). A higher prevalence of CI reporting was associated with reporting of results from randomized clinical trials and the requirement to report according to guidelines (eg, 522 of 574 [90.9%] in *Lancet Oncology*).

**Conclusions and Relevance:**

These findings suggest that the reporting style of statistical inference in abstracts of major cancer journals has improved over time. A requirement in journals’ instructions for authors to present statistical inference in accordance with reporting guidelines and the implementation of these guidelines in submitted manuscripts on the part of journal editors may improve reporting.

## Key Points

**Question:**

How has the reporting of statistical inference in abstracts of major cancer journals evolved over time?

**Findings:**

In this serial cross-sectional study, reporting of confidence intervals increased over time in most journals, with most abstracts including confidence intervals from 2016 to 2020; however, the proportion of abstracts reporting statistical inference based solely on the terms *significant* or *nonsignificant* was 24% during this period. Reporting of results from randomized clinical trials and the requirement to report according to guidelines were associated with a higher prevalence of confidence interval reporting.

**Meaning:**

These findings suggest that the reporting of statistical inference in abstracts of major cancer journals has improved over time and may continue to improve with the implementation of reporting guidelines.

## Teaser

This cross-sectional study investigates the time trend of statistical inference and statistical reporting style in the abstracts of major cancer journals.

## Introduction

Empirical studies usually include sampling, which is why the consideration of sampling error is important in the statistical estimation of associations or effects. In the 1920s, 2 competing schools of statistics developed: the null hypothesis test of Neyman and Pearson^1^ and Fisher’s significance test.^2^ These test-oriented methods have led to the widespread reporting of *P* values in current publications.

The *P* value combines information about the size of the effect and the size of the study so that the precision of the effect estimate cannot be judged by the effect size and *P* value alone.^3^ For this reason, researchers have required that a confidence interval [CI] be reported in addition to effect size since the 1970s.^4,5,6^ Since the 1990s, reporting guidelines such as Consolidated Standards of Reporting Trials (CONSORT) for randomized clinical trials (RCTs),^7,8^ Strengthening the Reporting of Observational Studies in Epidemiology (STROBE) for observational studies,^9^ and Preferred Reporting Items for Systematic Reviews and Meta-analyses (PRISMA) for systematic reviews and meta-analyses^10^ have been developed. Further reporting guidelines are provided by the Enhancing the Quality and Transparency of Health Research (EQUATOR) Network. The CONSORT, STROBE, and PRISMA reporting guidelines that apply to most empirical research among humans uniformly require that authors report a measure of precision (CI) in addition to effect size. Many journals currently require that these reporting guidelines be followed.

From this perspective, there is an order from most to least informative reporting of statistical inference^4^: (1) reporting CIs; (2) reporting *P* values, either as exact quantities (eg, *P* = .03) or as thresholds (*P* ≤ .05), without CIs; and (3) reporting only qualitative expressions (significant or not significant) without *P* values and without CIs. The aim of our serial cross-sectional study was to take advantage of the capabilities of PubMed to investigate the time trend of statistical inference and statistical reporting style in abstracts of major cancer journals.

## Methods

### Data Collection

In this cross-sectional study, we selected cancer journals according to their impact factor in 2019. We excluded the 3 journals with the highest impact factor—*CA: A Cancer Journal for Clinicians* (292.3), *Nature Reviews Clinical Oncology* (53.3), and *Nature Reviews Cancer* (53.0)—because these journals mainly publish reviews and educational articles. The 10 cancer journals in descending order of impact factor were *Lancet Oncology* (33.8), *Journal of Clinical Oncology* (33.0), *Cancer Discovery* (29.5), *Cancer Cell* (26.6), *JAMA Oncology* (24.8), *Annals of Oncology* (18.3), *Molecular Cancer* (15.3), *Journal of Thoracic Oncology* (13.4), *Journal of the National Cancer Institute* (11.6), and *Trends in Cancer* (11.1). With the exception of the *Journal of Clinical Oncology* and *Journal of the National Cancer Institute*, none of these journals was indexed earlier than 1990 in PubMed. We therefore restricted all time trend analyses to the period from January 1, 1990, to December 31, 2020. For the time trend analyses, we excluded 2 journals with only a few years of existence (*JAMA Oncology* and *Trends in Cancer*). Because we analyzed abstracts of publicly available articles in major cancer journals, there was no need for a review by an ethics committee. This study followed the Strengthening the Reporting of Observational Studies in Epidemiology (STROBE) reporting guideline.

To identify abstracts reporting results from RCTs, we used 2 criteria: *randomized controlled trial* (publication type) and the occurrence of the term *randomized* or its variants in the abstract text. The second criterion was necessary because otherwise post hoc analyses and other embedded projects within RCTs would be included in RCTs that were no longer RCTs themselves.

We searched PubMed for all abstracts of the 10 selected journals. We used a previously developed and validated search algorithm programmed in SAS, version 9.4 (SAS Institute, Inc) to identify the presence of CIs, exact *P* values (eg, *P* = .03) or comparisons of *P* values with thresholds (eg, *P* < .01), and significance terms.^11,12,13,14^ Previous validation studies of the search algorithm based on a random sample of 300 abstracts of clinical pharmacology journals published in 2012 to 2016^13^ revealed that the sensitivity of the detection was 95% for CIs, 98% for *P* value threshold, and 84% for exact *P* value reporting. Furthermore, the corresponding specificities were 100% for CIs, 97% for *P* value threshold, and 99% for exact *P* values.^13^

For this study, we drew a random sample of 100 abstracts across all major cancer journals and manually cross-checked the results of the search algorithm for the detection of *P* threshold, exact *P* value, significance terminology, and CI reporting. For these 4 characteristics, we detected 9 errors overall (9 of 400 = 2.3%). Eight of the 9 errors came from the well-known misuse of the term *significance* in a clinical sense rather than a statistical sense.^11,14^

### Statistical Analysis

Based on the 4 characteristics per abstract, we were able to categorize abstracts that contained any statistical inference as follows: (1) reporting of CIs, (2) reporting of *P* values (either as exact quantities or as thresholds) without CIs, and (3) significance terms only without *P* values and without CIs. For each publication year, we calculated proportions of the 3 reporting styles overall and by journals for abstracts. We calculated these proportions overall and stratified by RCTs for the most current period (2016-2020). We estimated time trends using weighted nonparametric local regression smoothing.^15,16^ Because we conducted a full survey of all abstracts from the selected journals from 1990 to 2020, we did not perform sample size or power calculations.

## Results

We reviewed 42 509 abstracts, 24 034 (56.5%) of which contained statistical inference. The total number of abstracts reviewed depended on the publication period. Journals with short publication histories (*JAMA Oncology* [n = 991] and *Trends in Cancer* [n = 517]) contributed only a few hundred abstracts. The instructions for authors of the journals as of October 10, 2021, differed with respect to the requirement of adherence to reporting guidelines. Four journals mentioned CONSORT, STROBE, and PRISMA (*Annals of Oncology*, *JAMA Oncology*, *Journal of the National Cancer Institute*, and *Lancet Oncology*). Another 3 journals mentioned only CONSORT (*Cancer Cell*, *Journal of Clinical Oncology*, and *Molecular Cancer*), and 3 journals mentioned none of these 3 guidelines (*Cancer Discovery*, *Journal of Thoracic Oncology*, and *Trends in Cancer*). The proportion of abstracts containing statistical inference varied from 40 of 517 (7.7%) in *Trends in Cancer* to 3468 of 4831 (71.8%) in the *Journal of the National Cancer Institute*. The very small proportion of abstracts with statistical inference in *Trends in Cancer* prompted us to manually review a random sample of 50 abstracts for which the algorithm indicated that no statistical inference was reported. Manual inspection confirmed this in all 50 abstracts (Table 1).

**Table 1.** Proportion of Abstracts in High-Ranking Cancer Journals Containing Statistical Inference

Journal	Origin	Period covered	Reporting guidelines^a^	No. of abstracts	Abstracts, No. (%)	
					Containing statistical inference	RCTs
All	NA	1990-2020	NA	42 509	24 034 (56.5)	5380 (12.7)
*Annals of Oncology*	Europe	1990-2020	1, 2, 3	8239	4760 (57.8)	1062 (12.9)
*Cancer Cell*	US States	2002-2020	1	2815	254 (9.0)	1 (0.04)
*Cancer Discovery*	US	2011-2020	None	2522	243 (9.6)	5 (0.2)
*JAMA Oncology*	US	2015-2020	1, 2, 3	991	689 (69.5)	146 (14.7)
*Journal of Clinical Oncology*	US	1990-2020	1	14 504	10 260 (70.7)	2876 (19.8)
*Journal of the National Cancer Institute*	US	1990-2020	1, 2, 3	4831	3468 (71.8)	391 (8.1)
*Journal of Thoracic Oncology*	US	2006-2020	None	3130	1894 (60.5)	194 (6.2)
*Lancet Oncology*	Europe	2000-2020	1, 2, 3	2467	1379 (55.9)	704 (28.5)
*Molecular Cancer*	Europe	2002-2020	1	2493	1047 (42.0)	1 (0.04)
*Trends in Cancer*	US	2015-2020	None	517	40 (7.7)	0

Abbreviation: RCT, randomized clinical trial.

^a^ Instructions for authors call for the consideration of Consolidated Standards of Reporting Trials (CONSORT [1]), Strengthening the Reporting of Observational Studies in Epidemiology (STROBE [2]), and/or Preferred Reporting Items for Systematic Reviews and Meta-analyses (PRISMA [3]) reporting guidelines.

The prevalence of statistical inference decreased in 4 journals (*Cancer Discovery*, *Journal of the National Cancer Institute*, *Journal of Thoracic Oncology*, and *Molecular Cancer*) since 2010, was more or less constant in 2 journals (*Cancer Cell* and *Journal of Clinical Oncology*), and increased in 2 journals (*Annals of Oncology* and *Lancet Oncology*) (eFigure 1 in the Supplement). The increase in prevalence during the period 1990 to 2020 was particularly large in *Lancet Oncology* (from 14.3% in 2000 to 70.9% in 2020) (eFigure 1 in the Supplement).

The prevalence of reports about RCTs over time showed an initial increase and then a decrease for the *Journal of the National Cancer Institute*. For *Annals of Oncology*, *Journal of Clinical Oncology*, and *JAMA Oncology*, the prevalence tended to increase in the most recent period. At *Lancet Oncology*, after an initial (2000-2004) prevalence of virtually zero, there was a rapid increase in prevalence to 46.7% in 2014, with a decrease thereafter. In *Cancer Cell*, *Cancer Discovery*, and *Trends in Cancer*, the prevalence of RCTs was zero or very low (eFigure 2 in the Supplement).

After exclusion of 2 journals with only short publication records (*JAMA Oncology* and *Trends in Cancer*), 5 of the remaining 8 journals showed increases in the prevalence of CI reporting (*Annals of Oncology*, *Journal of Clinical Oncology*, *Journal of the National Cancer Institute*, *Journal of Thoracic Oncology*, and *Lancet Oncology*). The increase started in the early 1990s in *Annals of Oncology, Journal of the National Cancer Institute*, and *Journal of Clinical Oncology*, whereas it appeared in *Lancet Oncology* for the first 10 years of its existence (2000-2010) and in the *Journal of Thoracic Oncology* in the recent 10 years (2011-2020). These increases were accompanied by a decrease in reporting of *P* values without CIs and significance terminology without *P* values and without CIs. The prevalence of reporting of statistical significance showed a steady increase only in the journal *Molecular Cancer*. Few journals showed little change in their reporting style of statistical inference in their abstracts, including *Cancer Cell* and *Molecular Cancer,* that both of which were dominated by reporting statistical significance only (Figure and eFigure 3 in the Supplement).

**Figure.**
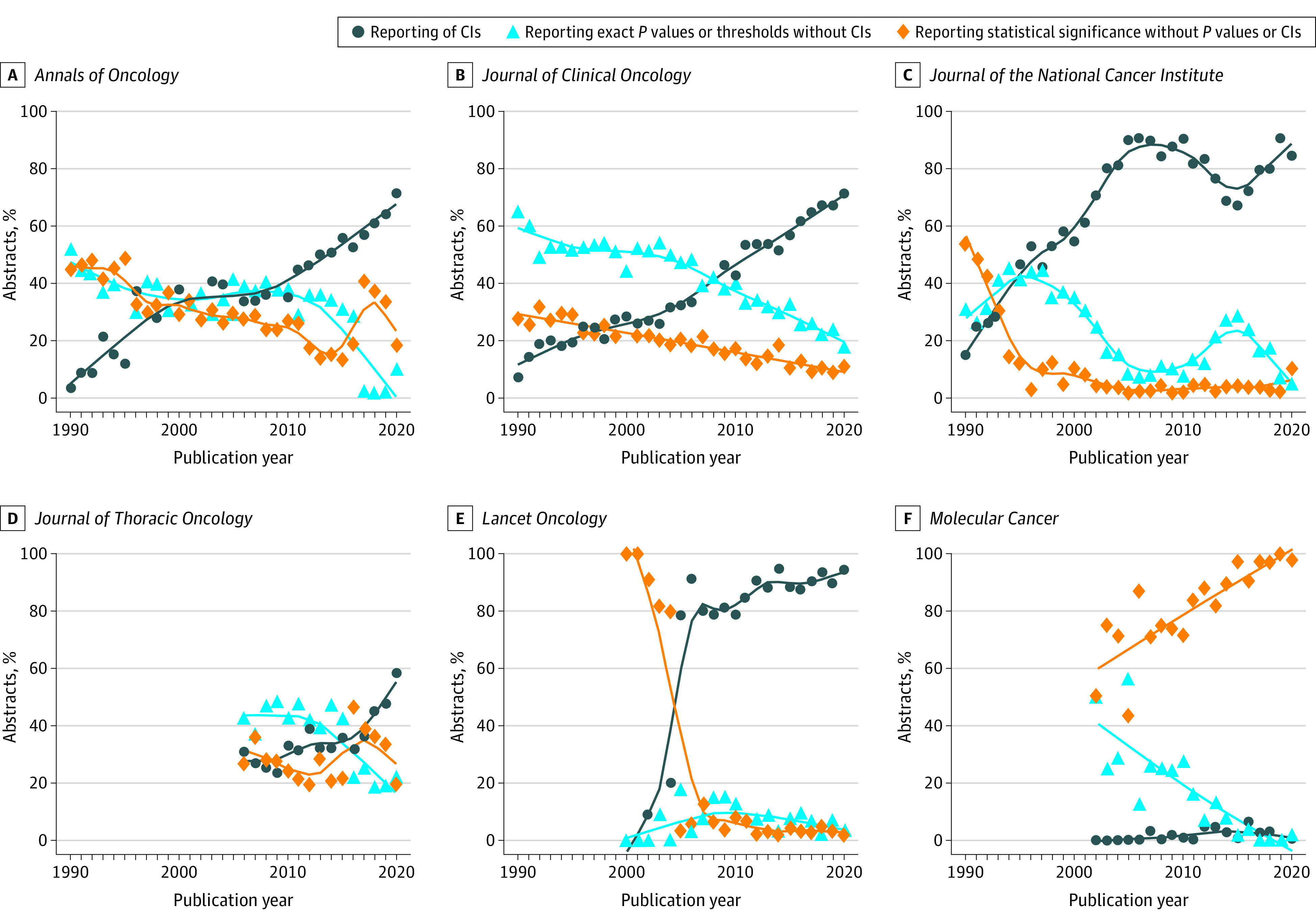
Flexibly Estimated Time Trends in 1990 to 2020 of the Statistical Reporting Style in Abstracts of Cancer Journals That Contain Statistical Inference Results are shown for 6 of the 10 cancer journals with high impact factors. All trend lines are smoothed using weighted nonparametric local regression smoothing. Results for the remaining 4 journals are displayed in eFigure 3 in the Supplement.

A comparison of journals for the most recent period (2016-2020) revealed that the percentage of abstracts containing statistical inference differed markedly by journals (34 of 490 [6.9%] in *Trends in Cancer* to 1338 of 1813 [73.8%] in *Journal of Clinical Oncology*). Among abstracts containing statistical inference, the percentage of RCTs was highest for *Lancet Oncology* (289 of 574 [50.3%]), followed by *Annals of Oncology* (212 of 752 [28.2%]), *Journal of Clinical Oncology* (348 of 1338 [26.0%]), and *JAMA Oncology* (126 of 616 [20.5%]). In particular, the more basic science–oriented cancer journals such as *Cancer Cell* (1 of 66 [1.5%]), *Cancer Discovery* (1 of 146 [0.7%])*,* and *Molecular Cancer* (0 of 284) rarely included reports on RCTs. The prevalence of CI reporting was high among those journals that frequently reported on RCTs (eg, 522 of 574 [90.9%] for *Lancet Oncology*). In contrast, the least informative reporting style—that is, reporting only whether an association was significant or not—was most prevalent among the basic science–oriented cancer journals (eg, 63 of 66 [95.5%] in *Cancer Cell*) and was found in 1195 of 4895 abstracts (24.4%) among all journals. Among the 3 reporting styles, reporting *P* values without CIs played only a minor role. The highest percentages of CI reporting were found in journals that call for consideration of all 3 reporting guidelines (CONSORT, STROBE, PRISMA), that is, *JAMA Oncology* (514 of 616 [83.4%]), *Journal of the National Cancer Institute* (473 of 580 [81.6%]), and *Lancet Oncology* (522 of 574 [90.9%]) (Table 2).

**Table 2.** Prevalence of Reporting of Statistical Inference in Abstracts of High-Ranking Cancer Journals From 2016 to 2020

Journal	Total, No.	Any statistical inference, No. (%)	Among abstracts containing statistical inference, No. (%)			
			RCTs	CIs	*P* values without CIs	Significance terms only
All	10 227	4895 (47.9)	1061 (21.7)	3070 (62.7)	630 (12.9)	1195 (24.4)
*Annals of Oncology*	1267	752 (59.3)	212 (28.2)	447 (59.4)	75 (10.0)	230 (30.6)
*Cancer Cell*	851	66 (7.8)	1 (1.5)	1 (1.5)	2 (3.0)	63 (95.5)
*Cancer Discovery*	1610	146 (9.1)	1 (0.7)	7 (4.8)	9 (6.2)	130 (89.0)
*JAMA Oncology*	897	616 (68.7)	126 (20.5)	514 (83.4)	9 (1.5)	93 (15.1)
*Journal of Clinical Oncology*	1813	1338 (73.8)	348 (26.0)	884 (66.1)	313 (23.4)	141 (10.5)
*Journal of the National Cancer Institute*	797	580 (72.8)	39 (6.7)	473 (81.6)	78 (13.4)	29 (5.0)
*Journal of Thoracic Oncology*	889	505 (56.8)	45 (8.9)	216 (42.8)	108 (21.4)	181 (35.8)
*Lancet Oncology*	824	574 (69.7)	289 (50.3)	522 (90.9)	34 (5.9)	18 (3.1)
*Molecular Cancer*	789	284 (36.0)	0	6 (2.1)	2 (0.7)	276 (97.2)
*Trends in Cancer*	490	34 (6.9)	0	0	0	34 (100)

Abbreviation: RCT, randomized clinical trial.

After subdividing abstracts between those reporting RCTs and all remaining abstracts, the prevalence of reporting CIs was higher for RCTs (849 of 1061 [80.0%]) than for other abstracts (2221 of 3834 [57.9%]). Reporting statistical significance alone occurred rarely in RCTs (70 of 1061 [6.6%] across all journals). In contrast, abstracts that did not report on RCTs showed a higher prevalence of using only significance terminology (1125 of 3834 [29.3%]) at the expense of CI reporting (eTable in the Supplement).

## Discussion

Overall, 24 034 (56.5%) of 42 509 abstracts contained statistical inference. Reporting of CIs increased over time in 5 of 8 journals. From 2016 to 2020, the most prevailing statistical reporting style was the presentation of CIs. However, the proportion of abstracts reporting statistical inference based solely on the terms *significant* or *nonsignificant* was still 24.4% from 2016 to 2020 and was most prevalent among basic science–oriented cancer journals. Reporting of results from RCTs and the requirement to follow reporting guidelines were associated with a higher prevalence of CI reporting.

The observed strong increase in the prevalence of any statistical inference in abstracts of *Lancet Oncology* starting in 2005 was accompanied by a sudden increase of the prevalence of abstracts about RCTs and is due to a major change in philosophy and scope. Until April 2005, *Lancet Oncology* was a review-only journal, but from May 2005 onward the journal accepted original research and review articles, which necessitated a change of editorial policies about data presentation and requirements (email communication with David Collingridge, PhD, editor-in-chief of *Lancet Oncology*, October 13, 2021). The very low proportion of abstracts with statistical inference in the journal *Trends in Cancer* is explained by the mission of this journal focusing on reviews, commentaries, and potential impact of basic, translational, and clinical findings.

It is unclear whether the increase in the prevalence of CI reporting was causally related to the publication of reporting guidelines such as CONSORT (1996), STROBE (2007), and PRISMA (2009). The increase started as early as the 1990s in the journals for which we could look back to 1990 (*Annals of Oncology*, *Journal of Clinical Oncology*, and *Journal of the National Cancer Institute*). The reporting style of statistical inference in a journal depends not only on the authors and the instructions, but also on how strongly the editor of the journal forces the authors to implement the instructions.^17,18^

Publishing practices regarding statistical inference differ between medical disciplines. A comparison of major cancer journals (2016-2020) with major psychiatric journals (2010-2015)^12^ and major cardiology journals (2017-2019)^14^ reveals that the proportion of abstracts containing statistical inference is lower in cancer journals (47.9%) compared with cardiology and psychiatric journals (59% and 52%, respectively). Compared with major psychiatric (26%) and cardiology journals (49%), the prevalence of CI reporting in cancer journals is higher (62.7%).

### Limitations

Several factors limit our results. First, the results of our abstract-based study are not necessarily generalizable to the results of a review of statistical reporting in full reports. Reading the full reports may show that authors use a different reporting style in abstracts than in the reports. Furthermore, the quantitative extent of statistical reporting as well as mixtures of different reporting styles can be determined. However, abstracts contain what authors and editors regard as the most important results, which justifies an isolated look at abstracts. Furthermore, many readers only read the abstract. Second, we only reviewed the 10 cancer journals with the highest impact factors as of 2019. It is difficult to speculate what the results of lower-ranking cancer journals would look like. Third, for time trend analyses of statistical reporting, some high-ranking cancer journals provided relatively short time series (*Cancer Discovery*, *JAMA Oncology*, and *Trends in Cancer*).

## Conclusions

The findings of this cross-sectional study suggest that the reporting of statistical inference in abstracts of major cancer journals has improved over time. The requirement in journal instructions for authors to present statistical inference in accordance with reporting guidelines and the implementation of these guidelines in submitted manuscripts on the part of journal editors may improve reporting.

## Notes

Supplement. **eTable.** Prevalence of Reporting of Statistical Inference in Abstracts of High-Ranking Cancer Journals of the Publication Years 2016-2020 **eFigure 1.** Flexibly Estimated Time Trends 1990-2020 in the Prevalence of Any Statistical Inference in the Abstracts of High-Ranking Cancer Journals **eFigure 2.** Time Trends of the Prevalence of Publications of Randomized Clinical Trials **eFigure 3.** Flexibly Estimated Time Trends 1990-2020 of the Statistical Reporting Style in Abstracts of High-Ranking Cancer Journals That Contain Statistical Inference
