# Supplementary material for: Reporting of Statistical Inference in Abstracts of Major Cancer Journals, 1990 to 2020
Source: JAMA Netw Open. 2022 Jun 23;5(6):e2218337. doi: 10.1001/jamanetworkopen.2022.18337 (PMC9227005; doi:10.1001/jamanetworkopen.2022.18337)
Supplement: Supplement. — eTable. Prevalence of Reporting of Statistical Inference in Abstracts of High-Ranking Cancer Journals of the Publication Years 2016-2020 eFigure 1. Flexibly Estimated Time Trends 1990-2020 in the Prevalence of Any Statistical Inference in the Abstracts of High-Ranking Cancer Journals eFigure 2. Time Trends of the Prevalence of Publications of Randomized Clinical Trials eFigure 3. Flexibly Estimated Time Trends 1990-2020 of the Statistical Reporting Style in Abstracts of High-Ranking Cancer Journals That Contain Statistical Inference [file jamanetwopen-e2218337-s001.pdf]

Supplementary Online Content

Stang A, Schmidt B. Reporting of statistical inference in abstracts of major cancer journals, 1990 to 2020. *JAMA Netw Open*. 2022;5(6):e2218337. doi:10.1001/jamanetworkopen.2022.18337

eTable. Prevalence of Reporting of Statistical Inference in Abstracts of High-Ranking Cancer Journals of the Publication Years 2016-2020

eFigure 1. Flexibly Estimated Time Trends 1990-2020 in the Prevalence of Any Statistical Inference in the Abstracts of High-Ranking Cancer Journals

eFigure 2. Time Trends of the Prevalence of Publications of Randomized Clinical Trials

eFigure 3. Flexibly Estimated Time Trends 1990-2020 of the Statistical Reporting Style in Abstracts of High-Ranking Cancer Journals That Contain Statistical Inference

This supplementary material has been provided by the authors to give readers additional information about their work.

© 2022 Stang A et al. *JAMA Network Open*.

eTable. Prevalence of Reporting of Statistical Inference in Abstracts of High-Ranking Cancer Journals of the Publication Years 2016-2020

**Journal**	**Total (n)**	**Any statistical inference n (%)**		**Percentages among abstracts containing statistical inference (%)**		
				**Confidence intervals**	**p-values without confidence intervals**	**Significance terminology only**
Randomized controlled trials (all journals)	1,126	1,061 (94.2)		80.0	13.4	6.6
Annals of Oncology	241	212 (88.0)		74.1	9.4	16.5
Cancer Cell	1					
Cancer Discovery	2					
JAMA Oncology	136	126 (92.6)		88.1	1.6	10.3
Journal of Clinical Oncology	361	348 (96.4)		69.8	27.6	2.6
Journal of the National Cancer Institute	40	39 (97.5)		92.3	7.7	0.0
Journal of Thoracic Oncology	53	45 (84.9)		80.0	2.2	17.8
Lancet Oncology	292	289 (99.0)		92.0	6.6	1.4
Molecular Cancer	0					
Trends in Cancer	0					

**Journal**	**Total (n)**	**Any statistical inference n (%)**		**Percentages among abstracts containing statistical inference (%)**		
				**Confidence intervals**	**p-values without confidence intervals**	**Significance terminology only**

© 2022 Stang A et al. *JAMA Network Open*.

Nonrandomized studies (all journals)	9,101	3,834 (42.1)		57.9	12.7	29.3
Annals of Oncology	1,026	540 (52.6)		53.7	10.2	36.1
Cancer Cell	850	65 (7.6)		1.5	3.1	95.4
Cancer Discovery	1,608	145 (9.0)		4.8	5.5	89.7
JAMA Oncology	761	490 (64.4)		82.4	1.4	16.3
Journal of Clinical Oncology	1,452	990 (68.2)		64.8	21.9	13.3
Journal of the National Cancer Institute	757	541 (71.5)		80.8	13.9	5.4
Journal of Thoracic Oncology	836	460 (55.0)		39.1	23.3	37.6
Lancet Oncology	532	285 (53.6)		89.8	5.3	4.9
Molecular Cancer	789	284 (36.0)		2.1	0.7	97.2
Trends in Cancer	490	34 (6.9)		0.0	0.0	100.0

© 2022 Stang A et al. *JAMA Network Open*.

eFigure 1. Flexibly Estimated Time Trends 1990-2020 in the Prevalence of Any Statistical Inference in the Abstracts of High-Ranking Cancer Journals

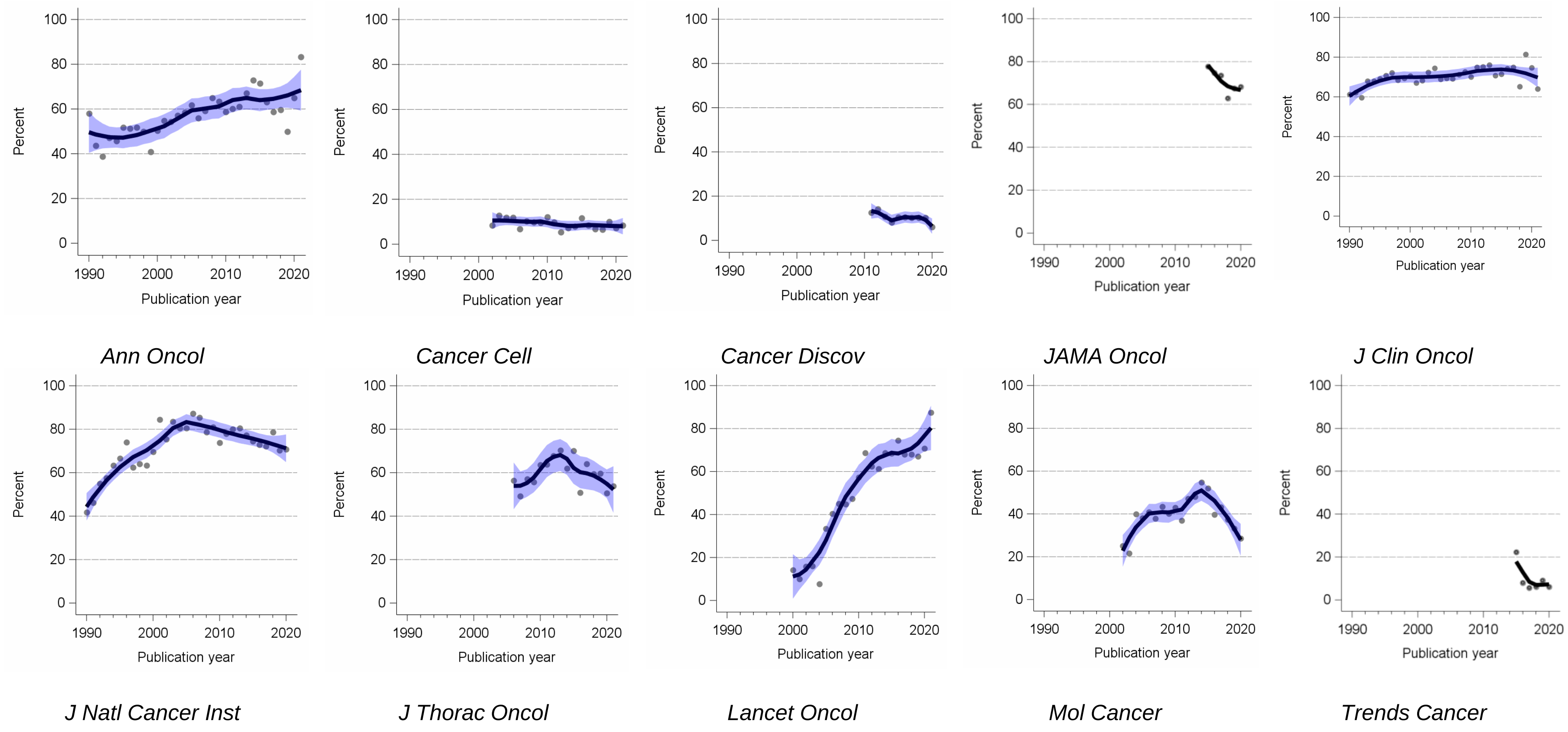

Legend: trend lines and 95% confidence interval bands are LOESS smoothed; no confidence interval bands for *JAMA Oncology* and *Trends in Cancer* because of few data points.

© 2022 Stang A et al. *JAMA Network Open*.

eFigure 2. Time Trends of the Prevalence of Publications of Randomized Clinical Trials

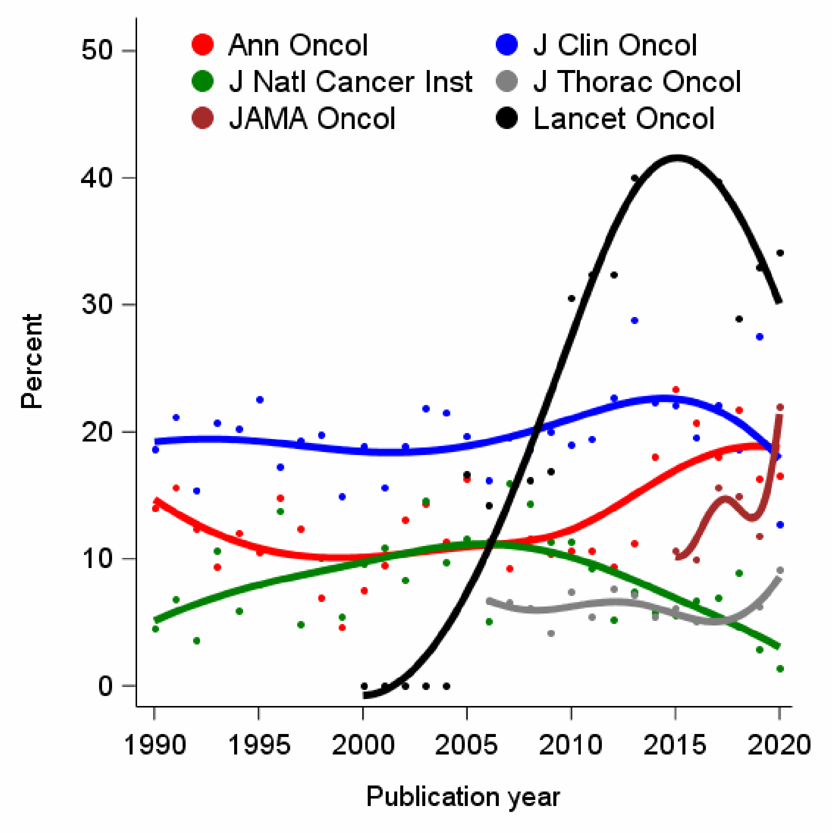

Legend: trend lines were based on splines (5 knots, smoothing 0.1)

© 2022 Stang A et al. *JAMA Network Open*.

eFigure 3. Flexibly Estimated Time Trends 1990-2020 of the Statistical Reporting Style in Abstracts of High-Ranking Cancer Journals That Contain Statistical Inference

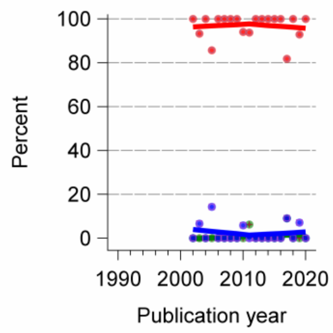

Cancer Cell

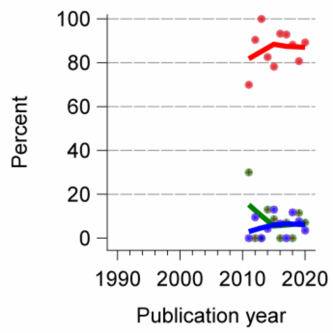

Cancer Discov

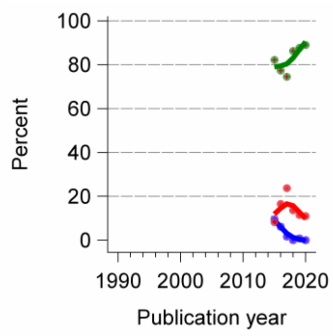

JAMA Oncol

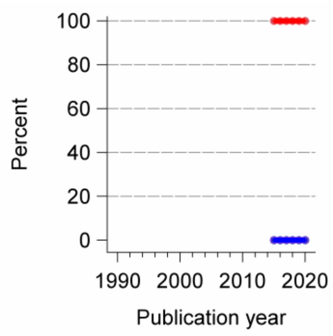

Trends Cancer

Legend eFigure 3: green graphs indicate reporting of confidence intervals; blue graphs indicate reporting of exact p-values and/or p thresholds without confidence intervals; red graphs indicate reporting of statistical significance without confidence intervals and p-values; all trend lines are LOESS smoothed

© 2022 Stang A et al. *JAMA Network Open*.
